# Severe Hyponatremia Associated With Terlipressin Use in Esophageal Variceal Bleeding

**DOI:** 10.7759/cureus.64576

**Published:** 2024-07-15

**Authors:** Fatima AlKindi, Omar AlHaj, Amnah Alhanaee, Raya Almazrouei, Yousef Boobes

**Affiliations:** 1 Division of Internal Medicine, Tawam Hospital, Al Ain, ARE; 2 Department of Gastroenterology, Tawam Hospital, Al Ain, ARE; 3 Department of Endocrinology, Tawam Hospital, Al Ain, ARE; 4 Department of Internal Medicine, College of Medicine and Health Sciences, United Arab Emirates University, Al Ain, ARE; 5 Department of Nephrology, Seha Kidney Care, Al Ain, ARE

**Keywords:** acute variceal bleeding, syndrome of inappropriate secretion of antidiuretic hormone (siadh), cirrhosis liver, terlipressin, acute hyponatremia

## Abstract

Terlipressin is an analogue of vasopressin that is indicated as first-line therapy for variceal hemorrhage and hepatorenal syndrome. Hyponatremia is an uncommon complication of terlipressin because it has less effect on vasopressin V2 receptors located in the kidneys. Profound hyponatremia related to terlipressin use is a rare complication that needs to be aware of. We described a 35-year-old previously healthy man, who was admitted for esophageal variceal bleeding that was attributed to hepatitis B-related liver cirrhosis. He had a normal baseline sodium level (Na 139 mmol/L) and developed severe hyponatremia 119 mmol/L (euvolemic, hypo-osmolar) at 72 hours of terlipressin therapy. After holding the medication, the hyponatremia corrected rapidly to 135 mmol/L within 24 hrs. Terlipressin was given again as therapy for overcorrection of hyponatremia and the sodium level decreased before being stabilized without neurological consequences. Severe hyponatremia is an uncommon complication of terlipressin therapy; however, our case emphasizes the importance of sodium monitoring during terlipressin therapy in all patients to prevent this complication, and more importantly, to avoid rapid correction that could happen after holding it.

## Introduction

Terlipressin is recommended as a first-line therapy for variceal hemorrhage and hepatorenal syndrome along with albumin replacement [1]. It is an analogue of vasopressin, with a strong effect on vasopressin V1 receptors leading to splanchnic vasoconstrictors and improvement of clinical condition [2]. It has a partial effect on renal vasopressin V2 receptors that can result in hyponatremia. Hyponatremia is classified based on the sodium level and clinical symptoms as mild (135-130 mmol/l), moderate (129-125 mmol/l), and severe hyponatremia (below 125 mmol /l). The risk for hyponatremia in patients treated with terlipressin varied from 6 to 19% [3,4]. The reported potential risk factors for terlipressin-induced hyponatremia include normal baseline sodium, usage of a higher dose of terlipressin, low model for end-stage liver disease (MELD) score, young patients, and terlipressin use for active variceal bleeding. However, severe hyponatremia is a rare complication that needs to be monitored. We report a 35-year-old male who developed terlipressin-induced severe hyponatremia during the management of variceal bleeding. 

## Case presentation

A 35-year-old male, previously healthy, had a sudden onset of epigastric abdominal pain and repeatedly a large amount of bloody vomiting one day prior to hospitalization. He also had dark tarry stool (melena) for two days and dizziness with poor oral intake. A review of the system was negative for fever, diarrhea, change in mental status, confusion, or urinary symptoms. His past medical, surgical, and family histories were unremarkable for gastrointestinal pathologies or bleeding diathesis. He was not on any regular medications or supplements. He denied alcohol intake and smoking.

In the emergency department (ED), his vitals were within the normal limits including a blood pressure of 140/88 mmHg, a heart rate of 70 beats/minute, a respiratory rate of 17 breaths/minute, and a tympanic temperature of 36.8 °C. His body mass index (BMI) was 28 kg/m^2^. He was pale and in mild distress but alert and oriented. He did not have jaundice or flapping tremors. Abdominal examination revealed coarse liver edge, normal spleen size, and no ascites or lower limb edema. Other systems' physical examinations were unremarkable. Initial laboratory investigations showed anemia with a hemoglobin of 82 g/L, thrombocytopenia (platelets of 124 x10^9/L), and normal white blood cell count (8.1 x10^9/L). He had a normal renal function test with a sodium level (Na) of 139 mmol/L. His liver enzymes and coagulation profile were within the normal range (Table 1).

**Table 1:** Laboratory investigations of the patient

Laboratory	Results	Normal range
Sodium	139 mmol/L	136- 145 mmol/L
Potassium	4.4 mmol/L	3.2-5.5 mmol/L
Chloride	109 mmol/L	98-107 mmol/L
Bicarbonate	22 mmol/L	22-29 mmol/L
Creatinine	60 micromol/L	62-106 micromol/L
Urea	9.40 mmol/L	2.80-8.10 mmol/L
Albumen	28 g/L	35-52 g/L
Bilirubin total	12.2 micromol/L	<21 micromol/L
Alkaline phosphatase	4.3 micromol/L	40-129 IU/L
Gama (GT)	63 IU/L	
Aspartate transaminase (AST)	26 IU/L	<40 IU/L
Alanine transaminase (ALT)	28 IU/L	<40 IU/L
HbA1c	7.6 %	4.4-6.4%
Thyroid-stimulating hormone (TSH)	3.170 milli IU/L	
T4 free	13.4 pmol/L	
White blood cell (WBC)	8.1 x10^9/L	4.5-11x10^9/L
Hemoglobin	90 g/L	132-173 g/L
Platelet	124 x10^9/L	140-400x10^9/L
International normalized ratio (INR)	1.12	
Aptt	20.5 sec(s)	
D dimer	1.620 mg/L	
Human immunodeficiency virus (HIV)	Non Reactive	Negative
Hepatitis BS Ag	Reactive	Negative
Hepatitis Be Ab	Reactive	Negative
Hepatitis HBV Core Ab	Reactive	Negative
MDX HBV PCR	62 IU/mL	Negative
Hepatitis C Ab	Non-reactive	Negative
Antinuclear antibodies (ANAs)	Negative	Negative
Antimitochondrial antibodies (AMAs)	Negative	Negative
Smooth muscle antibodies (SMAs)	Negative	Negative
Liver kidney microsome type 1 (anti-LKM-1) antibodies	Negative	Negative
Alpha-fetoprotein (AFP)	1.63 IU/mL	Negative
Cortisol level	301.0 nmol/L	
Ceruloplasmin	0.20 g/L	
Alfa 1 antitrypsin (A1-AT)	1.47 g/L	Negative
Ferritin	34 mcg/L	30-400mcg/L

**Figure 1:**
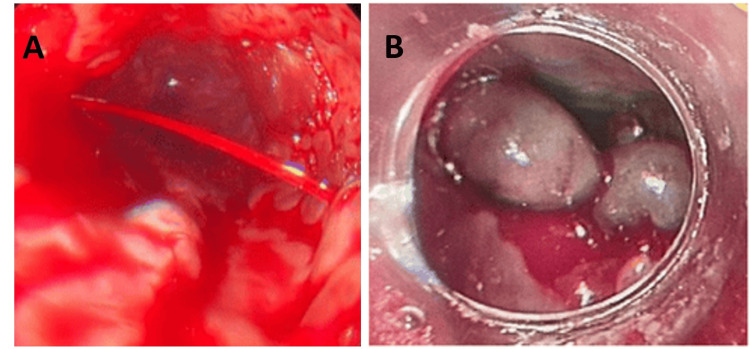
Esophagogastroduodenoscopy (A) Cherry red spots, actively bleeding third-degree esophageal varices; (B) varices managed by ligating bands

Initial management in the ED included one liter of normal saline, intravenous (IV) pantoprazole 40 mg twice daily, and transfusion of two units of packed red blood cells. Subsequent esophagogastroduodenoscopy showed actively bleeding stage III esophageal varices managed by eight ligating bands (Figure 1). 

He was started on Terlipressin 2 mg Q6hrs IV, IV albumin 20% 100ml, and IV ceftriaxone dose 2000mg twice daily. Ultrasound abdomen showed liver cirrhosis (without focal lesions) and portal hypertension. 

He was admitted to a high-dependency unit as a case of esophageal varices bleeding in newly diagnosed liver cirrhosis (CHILD B, MELD Na 8) for further management. His initial sodium level on admission was normal 139 mmol/L, and within 24 hours of terlipressin therapy, the sodium level reduced to 135 mmol/L. After 48 hours, his blood pressure was noted to be on the higher side 150/90 and the terlipressin dose was reduced to 1 mg IV Q6hr; however, sodium decreased further to 125 mmol/L. At this stage, the workup of the hyponatremia revealed a serum osmolality of 274 mOsml/kg, a urine Na level 103 mmol/L, and an elevated urine osmolality of 558 mOsm/Kg along with normal cortisol and thyroid function results. The lab findings were suggestive of hypo-osmolar, euvolemic hyponatremia due to syndrome of inappropriate antidiuretic hormone secretion (SIADH) due to terlipressin use.

**Figure 2:**
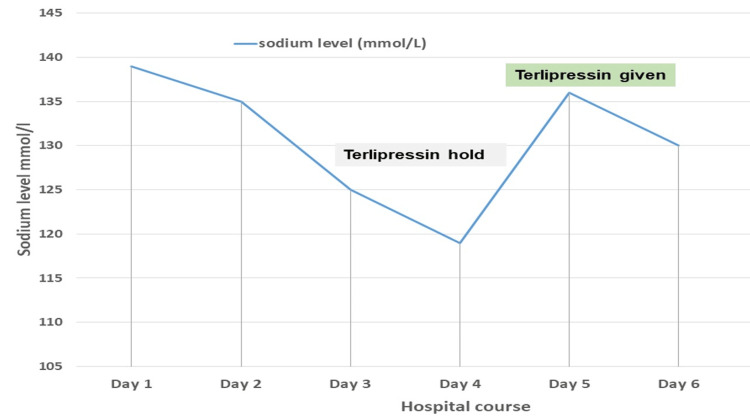
Changes in the serum sodium level with terlipressin use

With continued use of a lower dose of terlipressin 1 mg IV Q6hr (at 72 hours from initiating the therapy), he had severe hyponatremia of 119 mmol/L with mild symptoms of nausea and fatigue. At this point, terlipressin was stopped and a total of 210 ml (rate of 30ml/hr) of 1.8% saline was given. There was a rapid correction of the sodium level from 119 mmol/l to 126 mmol/L within eight hours, so 1.8% saline was held and the patient was advised to increase water intake. Despite these measures, the sodium level was rapidly overcorrected to 136 mmol/L within 24 hours. To minimize the overcorrection of hyponatremia, two doses of Terlipressin 1 mg q 12 hours were given, and he was started on IV D5% water and was instructed to increase fluid intake (2.2 liters fluid intake/24 hrs). The sodium level was carefully monitored and the repeated level at four hours was 130 mmol/L (Figure 2).

His clinical condition improved and the sodium level gradually increased to the normal range. On follow-up, the patient did not develop any neurological signs of osmotic demyelination syndrome.

An extensive workup conducted to identify the etiology of liver cirrhosis revealed chronic active hepatitis B virus infection (HBV quantitative PCR 361 Copies/ml, 62 IU/mL) and entecavir 0.5 mg therapy was initiated (Table 1). His clinical condition improved and he was discharged after 11 days of hospitalization. 

## Discussion

Early management of variceal bleeding with a combination of endoscopic therapy and splanchnic vasoconstrictors such as terlipressin resulted in better clinical outcomes in patients with cirrhosis. The European Association for the Study of the Liver (EASL) guidelines recommend fluid resuscitation, blood transfusion threshold of 7 g/dl, use of vasoactive drug therapy, antibiotic prophylaxis (ceftriaxone), and endoscopic therapy during active variceal bleeding in cirrhotic patients. The recommended dose of terlipressin is 2 mg every four hours during the first 48 hours, followed by 1 mg/4 hours for a total duration of 3 to 5 days depending on the clinical condition [5].

Terlipressin (1-triglycil-8-lysine-vasopressin) is a synthetic analogue of natural hormone vasopressin with a long half-life and relatively few side effects ranging from hypertension, hypovolemia, to hyponatremia. It acts mainly as a selective V1 receptor agonist with a small effect on the V2 receptor. Therefore, it has potent vasoconstrictor effects on splanchnic circulation via its action on the vasopressin V1 receptor in smooth muscles (mediated by the phospholipase C activation pathway). In renal collecting ducts, the activation of V2 receptors via the cAMP pathway results in increasing water reabsorption via aquaporins leading to a risk of hyponatremia [2,6]. The risk of hyponatremia may be exacerbated by an increase in endogenous vasopressin during active bleeding and shock.

The estimated incidence of terlipressin-induced hyponatremia is variable. Early randomized control studies of using terlipressin in gastrointestinal bleeding reported lower episodes of hyponatremia ranging between 0 and 6%. Feu et al. reported five cases of terlipressin-induced hyponatremia among 80 patients (6.25%), and similarly, 4 out of 105 patients (3.81%) developed hyponatremia in the study by Escorsell et al. [3,4]. Moreover, a retrospective study of 58 patients reported that 39 patients (67%) developed hyponatremia of which, in 18 patients (31%) it was moderate hyponatremia from 135.7 to 128.3 mEq/L (reduction by 5 and 10 mEq/L) and in 21 patients (36%) it was severe from 137.2 mEq/L to 120.5 mEq/L (reduction > 10 mEq/L). The majority of patients had recovered from hyponatremia within four days after completing the course of terlipressin therapy. Neurological manifestations developed in three patients with severe hyponatremia including one case of osmotic demyelination syndrome. In multivariate analysis, the risk for hyponatremia was higher in patients with normal baseline sodium and a low MELD score [7]. In a multicenteric randomized clinical trial, the use of terlipressin was associated with hyponatremia when compared to somatostatin and octreotide groups during acute gastroesophageal variceal hemorrhage [8,9]. Also, Yim et al. studied the risk factors for terlipressin-induced hyponatremia in 151 patients (mean age, 55.1±11.8 y, 19.2% severe hyponatremia) and concluded that younger age, higher serum Na level, lower Child-Pugh score, lower body mass index, and a longer duration of terlipressin were potential risk factors for this complication [10]. Han et al. identified similar risk factors for terlipressin-induced hyponatremia in 44 patients with acute variceal bleeding [11].

The results of such studies were in line with the significant antidiuretic activity of terlipressin in cirrhotic patients due to renal V2 receptor stimulation. Also, there were a few case reports about terlipressin-induced severe hyponatremia that emphasized the importance of sodium monitoring during terlipressin therapy [12,13]. Careful monitoring of fluid balance and the risk of considerable fluid retention during terlipressin therapy might influence the degree of hyponatremia. Also, close monitoring of the sodium level during terlipressin therapy is fundamental. There is no consensus recommendation regarding the frequency of sodium level monitoring during terlipressin therapy; however, it is better to have a baseline sodium level, then at 48 hours, then daily until therapy is completed. 

In our case, terlipressin was used in the management of severe variceal bleeding as per guidelines. The patient experienced the side effects of hypertension and hyponatremia within 48 hours of therapy. He developed severe hyponatremia Na 119 mmol/l (SIADH) at 72 hours despite dose reduction. Holding the terlipressin resulted in rapid correction of hyponatremia and giving one mg of terlipressin every 12 hours for one day helped in correcting the rapid correction. Few cases used terlipressin therapy to manage rapid hyponatremia correction [2]. Our patient had many risk factors for terlipressin-induced hyponatremia as described in published literature [10,11]. Rapid correction of hyponatremia can lead to osmotic demyelination syndrome if not managed properly. 

## Conclusions

Severe hyponatremia is an uncommon complication of terlipressin therapy; however, our case emphasizes the importance of sodium monitoring during terlipressin therapy in all patients to prevent this complication, and more importantly, to avoid rapid correction that could happen after holding it. Larger prospective studies are needed to identify potential risk stratification scores for such complications and potential preventive strategies. 
